# Collective Reflective Equilibrium, Algorithmic Bioethics and Complex Ethics

**DOI:** 10.1017/S0963180124000719

**Published:** 2025-04-01

**Authors:** Julian Savulescu

**Affiliations:** Centre for Biomedical Ethics, Yong Loo Lin School of Medicine, National University of Singapore; Uehiro Chair in Practical Ethics, University of Oxford

## Abstract

John Harris has made many seminal contributions to bioethics. Two of these are in the ethics of resource allocation. Firstly, he proposed the “fair innings argument” which was the first sufficientarian approach to distributive justice. Resources should be provided to ensure people have a fair innings—when Harris first wrote this, around 70 years of life, but perhaps now 80. Secondly, Harris famously advanced the egalitarian position in response to utilitarian approaches to allocation (such as maximizing Quality Adjusted Life Years [QALYs]) that what people want is the greatest chance of the longest, best quality life for themselves, and justice requires treating these claims equally. Harris thus proposed both sufficientarian and egalitari an approaches. I will compare these approaches with utilitarian and contractualist approaches. I will provide a methodology for deciding amongst these (Collective Reflective Equilibrium). I will apply this to the allocation of ventilators in the pandemic (as an example) and create an ethical algorithm for their deployment. I describe the concept of algorithmic bioethics as a way of addressing pluralism of values and context specificity of moral judgement and policy, and addressing complex ethics.

## John Harris and the Value of Life

Everyone's life is different. We have different abilities, talents, length of life, quality of life and desires for our lives. The value of life for John Harris is not determined by objective features like its length or quality.

“it does not follow from the fact that some lives are more desirable in virtue of their objective features than others, that those with more desirable lives have more valuable lives [that is, are more worth saving].”^1^

Rather, the value of life is determined by the degree to which a person values their life. A person's life matters “not because it is *a life*, but because it is *someone's* life, because her life is an enterprise in which she has, and takes, an interest.”^2^ Life *per se* is not of value, but of value to the extent that a person values it.^3^

Of course, people value their own lives to different degrees. Some people who experience incurable suffering no longer value continuing to live and desire euthanasia. Their lives have ceased to have value for them. However to the degree that people value their lives equally (that is, they want to continue to live), their lives should have equal value, and they ought to be treated equally, according to John Harris.

## A Problem: Limitation of Resources

In 2001, I wrote an Editorial for the BMJ^4^ on the Independent Inquiries into Paediatric Cardiac Services at the Royal Brompton Hospital and Harefield Hospital alleging that children with Down Syndrome were discriminated against because they were inappropriately "steered away" from surgery for heart defects. The report recommended that: "The Trust's policies confirm clearly that people with a disability are entitled to, and will be accorded … the same rights of access to services as those without a disability; and that consultants should take the lead in implementing policies and influencing attitudes regarding equality of access…Access to services, and priority for treatment, should be determined only on the basis of clinical need."

Approximately 50% of children born with Down syndrome have congenital heart defects.^5^ Many of these, such as holes in the heart, can be corrected by surgery. It is discrimination to deny these children surgery based on their disability unless doctors judged their disability to be so severe as to make life not worth living. Some doctors did believe this^6^ into the 1990s but it is now roundly rejected.

Children with complex cardiac abnormalities, including those with Down Syndrome, sometimes also require cardiac transplantation. However, there is a severe shortage of organs for transplantation especially paediatric transplantation. Only about 1/3 of children who need heart transplantation receive one. This is rarely performed in Down Syndrome^7^ despite 1/775 live births in the US being for Down Syndrome^8^. Indeed a study in 2024 of major paediatric heart transplant centers in the US showed only 26 patients with Down Syndrome[DS] listed for heart transplant between 1992 to 2020.^9^ The authors concluded that in those 26 transplanted, “outcomes in children with DS selected for transplant listing are comparable to pediatric HTx recipients overall.”

As these data suggest, children with Down Syndrome are given lower if any priority. A Channel 4 edition of *Inside Out* reported one case of a child who was denied heart-lung transplantation because of her Down's syndrome. Her mother believed the medical argument was: "There are so few organs they're not going to waste one on my child."^10^

In many countries with legislation preventing discrimination, it is probably unlawful discrimination to fail to give children with Down Syndrome an equal chance of receiving a heart transplant on quality of life grounds. For example, The European Convention on Human Rights 1950 states: "Everyone's right to life shall be protected by law … No one shall be deprived of his right to life intentionally" and "The enjoyment of the rights and freedoms set forth in this Convention shall be secured without discrimination on any ground such as sex, race, colour … or other status."

According to John Harris' account of the value of life, children with Down Syndrome have lives of equal value to those without Down Syndrome and ought to have an equal chance of receiving a cardiac transplant. He has been a staunch advocate of equality of access, or equal treatment for equal need. Indeed, he argued that the use of Quality Adjusted Life Years to measure the health economic outcome of medical treatments is discriminatory against the aged (ageist), the disabled (ablist) and those with rare expensive diseases (economist).^11^

As Harris succinctly puts it, each rational person wants *for himself or herself* at least three things from health care: *(a)* the maximum possible life expectancy; *(b)* the best quality of life; and *(c)* the best opportunity or chance of getting both.^12^ This is a version of egalitarianism. The good or value of health care is giving each person (c).

There are limits, according to Harris. In *The Value of Life*, he argues for a “fair innings” approach to the allocation of life-saving resources, such as hearts. That is, each of us would be treated equally until our fair innings had been achieved (an innings is a period of batting in cricket until one is “out” or is not longer allowed to bat and score runs). At the time of his writing, this innings was mooted at 70, but it might now be 80 or 85. This was the first “sufficientarian” approach to distributive justice in health care.

Harris' egalitarianism speaks in favour of giving children with Down Syndrome and equal chance of receiving a heart transplant. This is not because he believes disability is neutral like hair colour (as some disability scholars argue^13^. Harris believes disability is bad^14^. But just as a short life can be valued by the individual as much as longer life is valued by a different individual, so too a disabled life can be valued as much by the individual as the life of able-bodied individual is valued by that individual.

While Harris' egalitarianism underpins the law and stated values of the National Health Service, it is not in fact what dictates treatment in the UK or most other countries. As Harris realises, treatment is provided on cost-effectiveness grounds, which is in practice the cost/QALY (Quality Adjusted Life Year). QALYs include consideration of probability of benefit, length of benefit and value of benefit (in terms of quality of life). This approach is essentially utilitarian. According to utilitarianism, the right act is the act which maximises utility, which in this case is the health benefit of medical treatment (measured in QALYs).

Indeed, even Courts take utilitarian considerations into account. In 1995, Jaymee Bowen (Child B) was denied a second bone marrow transplant for leukaemia on grounds of cost and low probability of success. Sir Thomas Bingham, Master of the Rolls, appealed to a principle of maximising benefit when he rejected the father's appeal: "Difficult and agonizing judgments have to be made as to how a limited budget is best allocated to the maximum advantage of the maximum number of patients." Indeed, the General Medical Council has stated that "the clinical team in determining priorities and the utilisation of the resources made available to them by the NHS is entitled to take into account the likely success of the treatment proposed."^15^

Indeed, there are more severe congenital conditions than Down Syndrome. Trisomy 18 is one. It results in more profound intellectual disability, more complex cardiac abnormalities and children very often do not live beyond childhood. According to egalitarianism, babies born with Trisomy 18 should be given equal chance of receiving a heart transplant. Indeed, any individual no matter how profound their impairments of cognition or consciousness from brain injury, dementia, or other cause should not only be given consideration but moreover *equal* consideration for life saving organs, unless they have had their "fair innings".

Should children with Down Syndrome or Trisomy 18 have an equal chance of receiving a heart transplant? How should limited resources like hearts be distributed? What is justice?

These are questions I have been struggling with for 30 years. John Harris has outlined a plausible, coherent and compelling egalitarian account of justice in health care. But it is also inconsistent with much medical practice, including funding of services in the NHS, and it conflicts with other ethical theories. How should we make progress where appear to be a variety of conflicting approaches to issues like justice in health care?

## The COVID-19 Pandemic: Egalitarianism or Utilitarianism?

Such issues came into sharper relief in the pandemic. At times, there were shortages of ventilators, vaccines, masks and other medical equipment. Italy was initially hard hit and doctors there proposed age limits in the allocation of ventilators − this was an example of Harris' “fair innings” argument. This proposal received a hostile response^16^.

The standard medical approach to allocation of life-sustaining treatment is “first come, first served.” This is egalitarianism with no limits − time becomes the lottery to decide who lives and who dies. It is psychologically more comfortable for doctors − it appears as if “no decision” is made. However, it will mean that people with very low chance of surviving are treated, and they may live very short lives of very poor quality.

In the UK, it was believed that limiting access to ventilators on the basis of age, even a fair innings, would be ageist and in contravention the Discrimination Act, though this was disputed^17^. Instead doctors invented the category of “frailty”^18^ which was in fact of proxy for probability of survival, length of survival and quality of life, that is, utility. Frailty was not mentioned as a “protected category” in UK law and so could be used as a basis of medical decision making.

Most countries in the West embrace pluralistic values, like both equality and utility^19^. This can be seen in the ambivalence of British policy, sometimes egalitarian, sometimes utilitarian. Harris' contributions have been immense − his critique of utilitarianism is sharp, comprehensive and illuminating. However, society appears to embrace plural values, including both utility and equality, and indeed such inclusion seems justifiable.

## A Way Forward to Embracing Plural Values: Collective Reflective Equilibrium

In recent years, I have begun to explore a pragmatic, practical and implementable approach to policy formation on contentious issues like organ or ventilator allocation. It is based on John Rawls'1950's paper, Reflective Equilibrium.^20^ When faced with a difficult issue, such as organ allocation, we should consider a range relevant cases and our intuitions (or preferences or values) about these cases. We should then apply a broad range (pluralism) of theories, principles and concepts to these cases. Where theoretical prescriptions diverge from intuitive prescriptions, we should try to bring these into alignment, or make them coherent, sometimes modifying the theories, sometimes modifying the intuitions. It is not exactly clear how we decide which to modify (and that is work to be done) but it will be some form rational alignment, seeking to identify the weight of reason. Rawls himself set some constraints on who are rational deliberators and how they should deliberate (who were informed of the relevant facts, willing to use logic and engage in rational dialogue, and had empathetic imagination of all those affected by the policy or action).

In the original version of reflective equilibrium, this process occurred at an individual or small group level involving rational deliberators. In Collective Reflective Equilibrium, we seek to identify public values. This can be done by interviews, citizen juries, surveys, machine learning or the use of novel massive online platforms such as that employed by the Moral Machine experiment which used a novel gamified online platform to obtain over 40 million preferences for the programming of driverless cars^21^.

## Saving the Greatest Number?

One example of collective reflective equilibrium in practice is in deciding whether to save the greatest number. John Taurek in his classic paper “Should the Numbers Count?”^22^ asks us to imagine this kind of example. Imagine you are in charge of a coastguard rescue vessel. A ship has overturned a violent storm is brewing. To the north is a life boat with one occupant. To the south is a life boat with 5 occupants. You only have time to go north or south before the storm will overturn the boats and drown the occupants.

Which direction do you go, north or south? Taurek argues treating people equally involves tossing a coin as this gives all 6 sailors an equal chance (50%) of living. This is also what Harris' egalitarianism require. If each person wishes to live as much, and values their life equally, then to treat them as equals is to give them an equal chance.

This egalitarian prescription to give everyone an equal chance is inconsistent with public values. In one survey we conducted, the ‘Intensive Care Life Boat' study, only 2 out 109 members of the public opted to toss a coin to decide whether to save one life or five; the other 107 all opted to save the greater number.^23^ In the Moral Machines experiment, saving the greatest number was the second strongest global preference (after saving humans rather than animals)^24^. Ordinary people believe we should save the greatest number.

Utilitarianism clearly directs us to save the 5 sailors (the greatest number). What about contractualism, a theory Rawls himself supported? A simplified version of Rawls' view would say that the morally just course of action is the one we would choose from behind a “veil of ignorance”, that is, if we did not know who we would be in the dilemma under consideration, whether it is the one sailor north or one of the five south. A policy of saving the greatest number would be in our rational self-interest. It would give 5/6 chance of living.

Both utilitarianism and contractualism converge with public values to prescribe saving the greatest number. This diverges from egalitarianism. The convergence of two dominant ethical theories together with coherence with public values makes saving the greatest number a highly plausible policy (at least compared to egalitarianism without public support). Of course, a more complete exercise of collective reflective equilibrium would not limit itself to 3 theories, but bring a wider range of theories and principles to bear.

## Priority for the Young?

Harris has been a prolific critic of ageism and discrimination against the elderly, especially through the use of QALYs^25^. However, another public value which we identified and which is the third strongest preference identified in the Moral Machines experiment, is priority for the young over the old. In our work, people strongly preferred to save younger lives than older lives, even when the length of future life was the same^26^.

This is in conflict with Harris' own fair innings approach − Harris would only give priority to lives below some threshold, and below that threshold (he stipulated 70), they would be given equal priority.

So which principle − priority to the younger or fair innings − should inform public policy?

Utilitarianism would favour saving the young as they will live for longer. It is for this reason Harris has argued that the use of QALYs to allocate resources in health are ageist.

According to contractualism, we should imagine we are behind a veil of ignorance, unaware of whom we will be. For example, if trying to decide whether to provide a ventilator or transplant to a 20 year old or a 60 year old, we should imagine whether we will be the 20 year old or the 60 year who need the limited life saving resource. Whom would we wish to be saved? It would be rational to prefer to save the 20 year old, because that individual will live roughly another 60 years. The 60 year old will only live roughly another 20 years. Contractualism again converges with utilitarianism to support the popular view that we should give priority to younger lives, rather than a fair innings.

As we argue in our paper elaborating Collective Equilibrium^27^, egalitarianism can be given a Kantian defense. And indeed in some societies, there is no public preference to save younger lives, such as East Asian societies^28^. Harris may well be able to marshal other defenders than Kant to support his egalitarianism, and there may be public support for his views in some parts of the world (eg Germans are typically more influenced by Kant, whereas the English are typically utilitarian, as a vast generalisation). I have not given some definitive argument in favour of priority for younger people, rather than a fair innings. What I have tried to do is begin to sketch how progress might be made by appealing to a range of theories as well as public intuitions in something like reflective equilibrium. Where it leads, who knows?

Contractualism and utilitarianism converge on saving younger lives when those younger people will live longer than older people. But what if they live equal amounts of time. Imagine we could save a 35 year old for 20 years, or a 50 year old for 20 years. Whom should we save?

Harris' fair innings argument would apply that they should be treated equally. But in our survey of allocation of ventilators, we found the public strongly supportive of saving the younger person, that is, the 35 year old in the example^29^. Utilitarianism would not support priority to the younger person in this example.

However, there is another reason to save the younger person, that would support saving the 35 year old for 20 years rather than the 50 year old for 20 years. That is *desert*. The younger person has had less of a good − life. So, they deserve more. Arguments from desert might also support giving priority to the younger person.

## Variable Value of Life?

For John Harris, the value of life is determined by the value people place on their own lives.

“The point is this: if we allow that the value of life for each individual consists simply in those reasons, whatever they are, that each person has for finding their own life valuable and for wanting to go on living, then we do not need to know what those reasons are. All we need to know is that particular individuals have their own reasons, or rather, simply, that they value their own lives.”^30^

“But where people do not in fact value their own lives or do not want their lives to continue, then of course it will not be wrong for them to kill themselves, or for others to help them do so, or for others to kill them at their request… for individuals by wishing to die show that they do not value life, or that they value death more. To frustrate the wish to die will on this view be as bad as frustrating the wish to live, for in each case we would be negating the value that individuals themselves put on their lives.”^31^

If we can value life not at all, presumably we also value life to different degrees, and we might value our own lives to different degrees at different ages. This opens up another possibility for why we should give priority to younger lives.

In one of my favourite pieces of bioethics literature, *Life's Uncertain Voyage*, Peter Singer argues life is like a voyage^32^. He imagines a person climbing a mountain in the Himalayas. In the first stage, the person has the vague idea of possibly climbing the mountain. As time progresses, the idea crystallizes and the desire becomes stronger until they begin preparations. Then follows the long journey to Nepal, and slowly beginning to climb the mountain. As they get closer and closer to the peak, their excitement and anticipation increase. At the peak, there is exhilaration and a deep sense of achievement. Then follows the long descent and the journey back home.

Singer argues that it is worst if the journey is aborted, or interrupted, right before the peak is ascended. It would be less bad if it were aborted when it was just a vague whim, or when on the journey home. Life's value, he argues, varies according to how close we are to our most important projects, ambitions and investments.

Martha Nussbaum gives similar arguments:

“… if one invests a lot of time in plans and hopes for the future, engaging in activities the whole point of which is preparatory (say, professional training), an unexpected death can make those activities vain and futile.”^33^

Ronald Dworkin introduces the related concept of ‘creative investment' in a life by the person themselves and others.

“The death of an adolescent girl is worse than the death of an infant girl because the adolescent's death frustrates the investments she and others have already made in her life − the ambitions and expectations she constructed, the plans and projects she made, the love and interest and emotional investment she formed for and with others, and they for and with her.”^34^

He argues that death is less bad as one gets older:

“But how bad this is- how great the frustration- depends on the stage of life in which it occurs, because the frustration is greater if it takes place after rather than before the person has made a significant personal investment in his own life, and less if it occurs after any investment has been substantially fulfilled, or as substantially fulfilled as is anyway likely.”^35^

This family of arguments would support giving greatest value to lives in the 30s or 40s, with life's value declining gradually on either side of that. That is, “creative investment” arguments might support giving priority to younger individuals, at least those with substantial creative investment.

Indeed, a version of Harris' own account might support giving priority to younger individuals. If younger individuals value their own lives more, the valuing life account might converge with the creative investment accounts. Harris does not go into what constitutes “valuing” much but if it were construed as “rational preferences” it might be that our strongest rational preferences for living would be as our creative investments reach their peak and are realised, much like reaching the peak in Singer's voyage.

What I have tried to show is that whether or not we should give some priority to saving younger lives is not settled by merely applying one plausible theoretical account, like the fair innings account, or simply by appeal to public values and intuitions. We require a consideration of broad range of theoretical approaches as well as public values and intuitions, and seek to bring these into maximum equilibrium. That is the task of bioethics.

## Collective Equilibrium and Deciding Health Policy

If we accept the principle that we should save the greatest number of people, this has implications for health policy and the allocation of limited resources. It implied, in the COVID-19 pandemic, that ventilators and vaccines (and other life saving resources) should have been allocated to those with the highest increment in chance of survival, and in the case of ventilators, those who would require ventilation for the shortest amount of time.

Consider increment in chance of survival first. Imagine there are two groups who will die without ventilation. Group A has a 20% chance of surviving and Group B an 80%. For every 10 people treated in each group with the resource, 2 people from Group A would be saved, and 8 people from Group B. We save more people if we direct resources to Group B.

Consider secondly length of treatment. Imagine there are two groups who will die without ventilation. Group C requires ventilation for 6 weeks and Group D for 2 weeks. Directing resources to Group D rather than C would save 3 times as many people.

On the basis of these considerations, Dominic Wilkinson and I have argued that one mandatory step in allocating ventilators when there is a shortage is to consider incremental probability of survival and duration of use of the resource^36^. Such a policy would save the greatest number. And we argued that this has the strongest public and theoretical support from a range of different theories, despite some versions of egalitarianism like Taurek's suggesting equal treatment requires something like a lottery (or the standard medical practice of “first come, first served”).

There are, of course, many other values which are relevant to allocation. For example, for utilitarians, it is not only number which is important, but also length and quality of life saved^37^. Utilitarians would also consider the maximization of QALYs. (Indeed, pure utilitarians would not consider saving the greatest number of people at all, but saving the greatest number of QALYs. What we described in our algorithm was a utilitarian step after an initial step of triage to save the greatest number − it is only partly utilitarian.)

After triage for probability of survival and duration of use, egalitarians might employ a lottery if resources are still insufficient for all. Some consideration might be given to responsibility, or social value. And some consideration might be accorded to historical injustice, or age. There are a range of possible policies.

**Figure 1:**
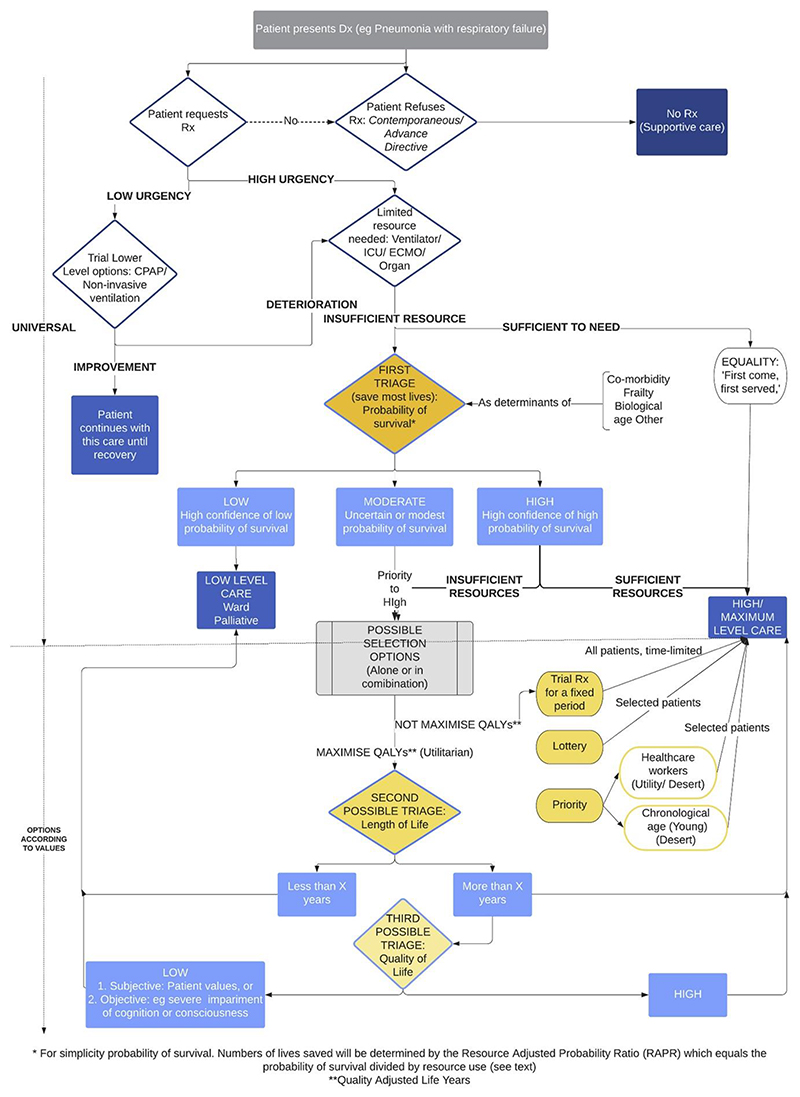
An Ethical Algorithm for Rationing Life Sustaining Treatment^38^

We encorporated some of these considerations into a decision algorithm which described of a mandatory initial triage considering probability of survival and duration of use of the resource, followed by different subsequent triages based on different value consideration. In principle, Collective Equilibrium could be used to decide amongst these different optional steps for a relevant population. We chose a mandatory stage of “saving the greatest number” as this was the strongest relevant preference in Awad et al's survey of 40 million preferences and we judged to be the least controversial value preference. See Figure 1

We did not attempt to create coherence between theoretical considerations (theories and principles) and public values, we did extensively survey public opinion pre-pandemic about allocation of intensive care beds^39^ and during the pandemic around allocation of ventilators and vaccines.^40, 41, 42, 43^

Across these studies, ordinary people preferred to save (in stark contrast to John Harris' own egalitarian views)

1. People who had a higher probability of survival
2. People who would live longer after being saved
3. People who were younger
4. People whose diseases were cheaper to treat or would use the resource for a shorter time(under conditions of limited money, equating to saving a greater number)
5. People who were abled rather than disabled

And they were prepared to withdraw ventilators from poorer prognosis patients for better prognosis patients^44^, which is rarely if ever done in medicine.

It was only when factors between different recipients of life saving interventions were very similar (e.g. 49% chance of survival vs 51%) did significantly more people become egalitarian, choosing to toss a coin (though even in these situations in many cases a substantial minority were still utilitarian).

Harris would, I suspect, strongly disagree with this approach. He argued the preferences revealed by the Moral Machines study should have no bearing on policy for programming driverless car. Instead, moral theory alone should inform policy. In characteristically swashbuckling style, he described the work in the Moral Machine experiment as ‘useless'.^45^ Harris argues that,

“The idea that it might be open to individual citizens or corporations to decide who shall be “spared” and who condemned to death, and that this might be a matter of mere individual “preference”, made on the basis of the sorts of sampling described in [the Moral Machines paper]… is outrageous in the extreme.”^46^

Here I agree with Harris, who writes that a solution to a moral dilemma “has to show how the circumstances which make it a moral dilemma, have been weighed carefully one against another, and morally persuasive reasons, facts and/or justifications found for having a moral preference for one outcome rather than another.” Importantly, however, this doesn't entail that public preferences have no role to play in the process—especially when its output is meant to be a practicable and implementable policy in a liberal democratic society. And we would need not only an ethical underpinning, but a converging ethical underpinning for these views, for example by seeking a convergence between utilitarianism and contractualism (as a veil of ignorance).

However, appealing to moral theories alone, as Harris suggests, is also problematic. First, there is no universally accepted moral theory − philosophers deeply disagree about whether to be Kantians or Utilitarians, for example. Although many moral theories embrace certain fundamental moral ideas (such as the equality), they interpret these ideas in radically different ways. Hence in Utilitarianism, equal moral consideration is expressed by counting each person's utility equally; in Kantian ethics, equal standing is understood to relate to our autonomy and dignity; on contractualist views, equal standing may relate to equality of position behind the veil of ignorance, while egalitarians call for equal treatment for equal need^47^.

Most plausibly, all or at least some of these considerations matter. Consider the basic conflict between utility and equality in the initial organ allocation example. It is plausible that both utility and equality matter^48^. This is expressed in the relatively modern conception of justice called “prioritarianism”^49^ where some priority is given to utility benefits to the worst off. “Justice (as purely equality), and let the Heavens fall” would be absurd − it would require directing all resources to the worst off until equality was achieved. And pure Utilitarianism would also be absurd − it would require taking organs from healthy individuals to save the lives of a larger number of people, the Survival Lottery which John Harris famously originally conceived as an objection to Utilitarianism^50^.

Or consider the inclusion of disability as consideration in the allocation of organs, as in the initial example. People are fiercely divided over whether disability should be considered in organ allocation and whether it constitutes unjust discrimination. Some will object that quality of life considerations or disability should not affect distribution of resources. Myopia, for example, is not relevant − we should not deny the short sighted (like me) organs because they need to wear glasses. However, extreme disability clearly should be a relevant consideration. Take the example of total disability: permanent unconsciousness^51^. No one would reasonably argue that we should give those who are permanently unconscious equal consideration for an organ transplant. Similar arguments apply to a minimally conscious state.

What about more widely discussed disabilities, like blindness, deafness and paralysis. Because these individuals have intact mental lives, and report equal levels of happiness after adaptation, there is a strong argument for equal consideration.

Ethics is not black and white; it black, white and grey. Many disabilities will be in the grey zone.

As soon as plural values are introduced into moral conversation and dialogue, people are likely to conclude (wrongly), “Ah, ethics is just relative, to culture, place, time or personal preference.” This is a deep mistake. It means that moral progress is not possible. It means that there is no strong or deep criticism of what the Nazis did − they just had different values. It makes Universal Declaration of Human Rights baseless, and international codes nearly meaningless.

While moral relativism is false, context dependency is true of moral judgements. Moral judgements *supervene* on the particular facts of situations. Even with the same values being considered, there will be different justified policies, moral judgements or prescriptions in different factual circumstances. To consider both utility and equality, with more resources, more consideration can be given to equality, and “grey zone” cases.

The best example of the context specificity of applying plural values is seen in consideration of the two values of liberty and public health. In the 1980s, Cyprus was bankrupting its health system and using all its blood supply in treating an “epidemic” of thalasseamia. Thalassaemia is a genetic disorder affecting production of blood cells, resulting in pain, deformity and premature death.

Carriage of the gene for thalassaemia is very high Cypriot populations. If both parents are healthy carriers, they have a 25% chance of having a child with the severe form of the disorder. Prenatal testing of fetuses and termination of affected pregnancies became possible in the 1980s.

One very important value which John Harris has consistently championed is procreative/reproductive autonomy, or procreative/reproductive liberty − the freedom to decide whether to have children, how many children to have, when and what kind of children. This is important western liberal societies. However, Cyprus was facing a public health emergency. In response, the Church decided to require people wishing to be married in the Church to have carrier testing. It did not require that they go to have prenatal testing, or termination of pregnancy. However, having witnessed the pain and misery of thalassaemia close up, nearly everyone who found they were carriers voluntarily chose prenatal testing and termination of affected pregnancies. The rates of thalassaemia plummeted. Problem solved.

This was “mandatory carrier testing”. It was a form of coercion and arguably some infringement in their procreative liberty. People were no longer free to get married in ignorance of their carrier status. But the level of coercion used by the Church was an effective and proportionate response to a public health crisis. Although both liberty and public health were important, some restriction of liberty was justified *in that circumstance*.

Of course libertarians, whose sole value is liberty, would object. But these monotheistic moral approaches just are not plausible.

## Algorithmic Bioethics

**Fig 2:**
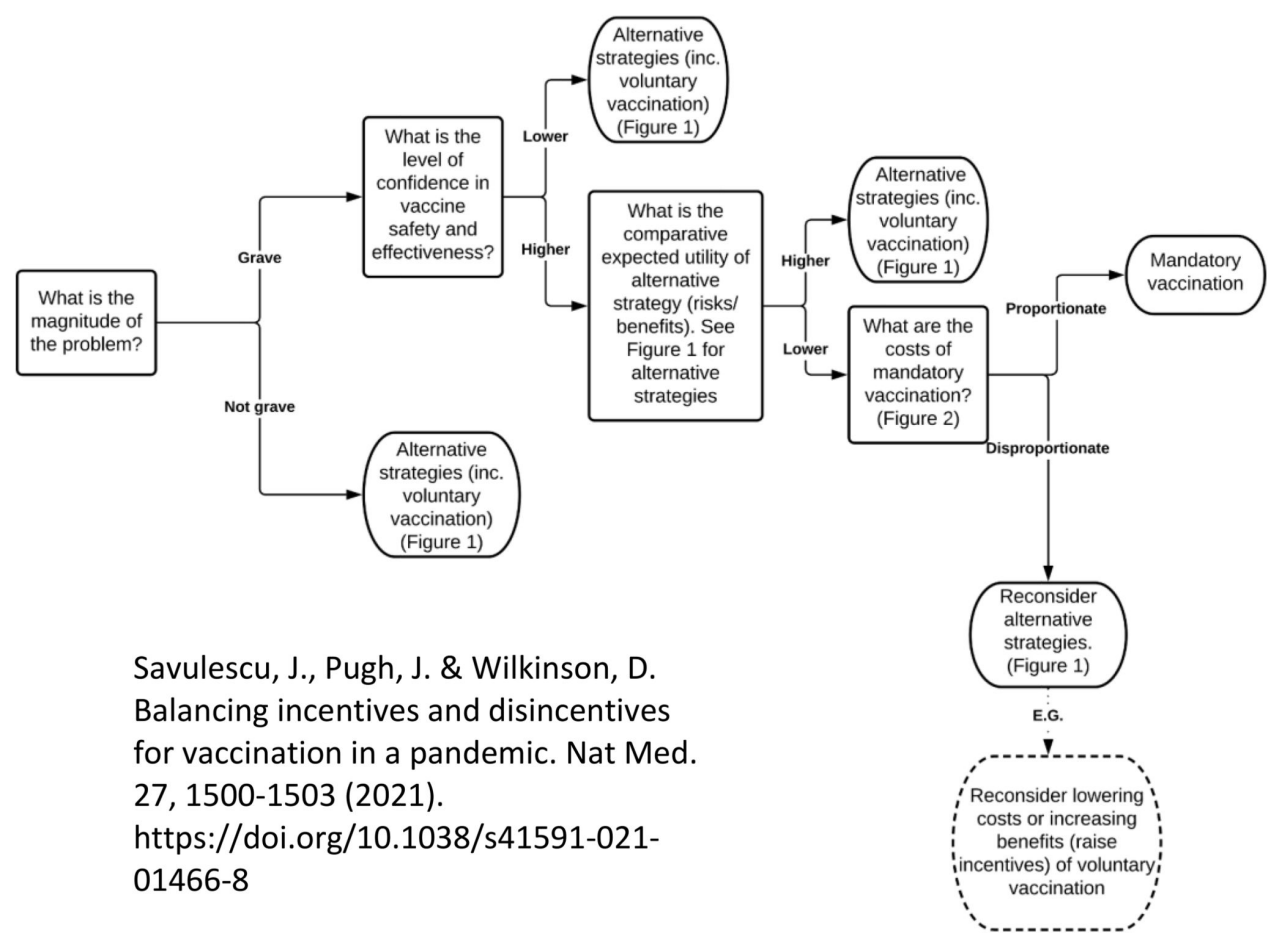
Algorithm for Mandatory Vaccination^52^

Early on in the pandemic, before vaccines for COVID-19 were developed, I identified the factors and values which would make mandatory vaccination justifiable (the same factors are relevant to all coercion): gravity of the problem, safety and effectiveness of vaccines, incremental utility of coercion over other less coercive measures, and proportionality of the costs of coercion to the incremental utility of coercive policies. I constructed an algorithm using these 4 factors. See Fig. 2. This is an example of algorithmic bioethics.

Algorithmic bioethics seeks to lay bare and make clear what the relevant values are to a policy. Further, it seeks to order these logically in a decision tree so they can be applied in practical situations. If someone disagrees with the inclusion or exclusion of a value, or their priority, they can amend the algorithm. The algorithm is clear (transparent) to all.

Importantly, the algorithm can be applied to the facts of a particular situation to yield a determinate prescription. When I applied my algorithm to the facts of COVID-19 (lethal primarily for the elderly, vaccines poor at preventing transmission, etc) I judged that mandatory vaccination was only justifiable for the elderly, over say 65^53^. Italy and Greece implemented such a “selective” mandatory vaccination policy by imposing fines on those over a certain age who refused vaccination.

Algorithmic bioethics is no silver bullet for practical ethics but it does enable progress and inclusion of diverse values like utility and equality.

However, for each value point in the algorithm, there will be further inquiry, debate and dialogue over how to interpret or specify that value. There may even be an algorithm behind that particular value to determine that value. For example, in the case of mandatory vaccination: what is grave public health emergency? What is safe and effective enough? What is proportionate coercion? We drafted a simple algorithm for how to determine whether coercion is proportionate^54^.

I have applied algorithmic bioethics to many other dilemmas in medical ethics: access to experimental treatment, resolution of conflict between parents and doctors^55^, determining futility^56^, when it is ethically justifiable to used biassed training data in AI^57^, vaccine passports, employing incentives and disincentives^58^, innovative care including AI (under consideration), and others.

I do not claim these algorithms are right or perfect. But they are clear basis for making progress because they can be revised or replaced. Bioethics needs to be clear, practicable and justifiable. And such algorithms can be polytheistic, instead of monotheistic, embracing plural values, like utility and equality.

Further, these decision tools can be used by policy makers and potentially programmed into AI, moral AI. AI has a vastly superior capacity compared to humans to harness facts about the world and people. This could be coupled with this human constructed ethical decision procedure to make ethical decisions. For example, pre-AI Dominic Wilkinson and I suggested the cost/QALY threshold could be applied consistently beyond new medicines and devices, to procedures and interventions, such as ventilation or surgery for intensive care patients^59^. (We did not endorse cost/QALY but rather argued that if it were an acceptable ethical way to distribute resources, it could be consistently applied to procedures.) AI could be used to retrieve the relevant data to calculate the cost-effectiveness of many more parts of the clinical pathway. Coupling AI with algorithmic bioethics could be ethically transformative for medicine.

## Life Extension

Another of John Harris' pioneering contributions has been to argue for life extension. But how long should we live (if there are sufficient resources)?

Harris argues that we have a moral duty to extend life as long as possible, even to immortality because saving a life and delaying death are equivalent. So if we have an obligation to save lives, we have an equal obligation to extend life indefinitely.^60^ Harris suggests it might be preferable to limit reproduction rather than the length of people's lives if overpopulation is a problem.

In recent unpublished research, together with Brian Earp and colleagues, we surveyed 198 members of the British public to see how they would choose between different possible future worlds to explore their values relating to length of life of future persons vs number of new lives in existence (what I called the length vs number trade off). People strongly preferred that there be fewer people with longer lives, rather than more people with shorter lives, even exponentially larger numbers of people. For example, 69% preferred a population of 2 million living 100 years compared to 31% who preferred a population of 64 million living 75 years.

But this preference began to flip at 150 years − that is, after people were living 150 years, they started to prefer there be more people rather than fewer people living even longer. For example, 52% preferred a world with a population of 2 million living 200 years and 48% preferred a world with a population of 64 million living 100 years. However, 36% preferred a population of 2 million living 200 years compared with 64% who preferred a population of 8 million living 150 years.

We asked them explicitly what principle should govern how long people should live. Here are the results:

**Figure:**
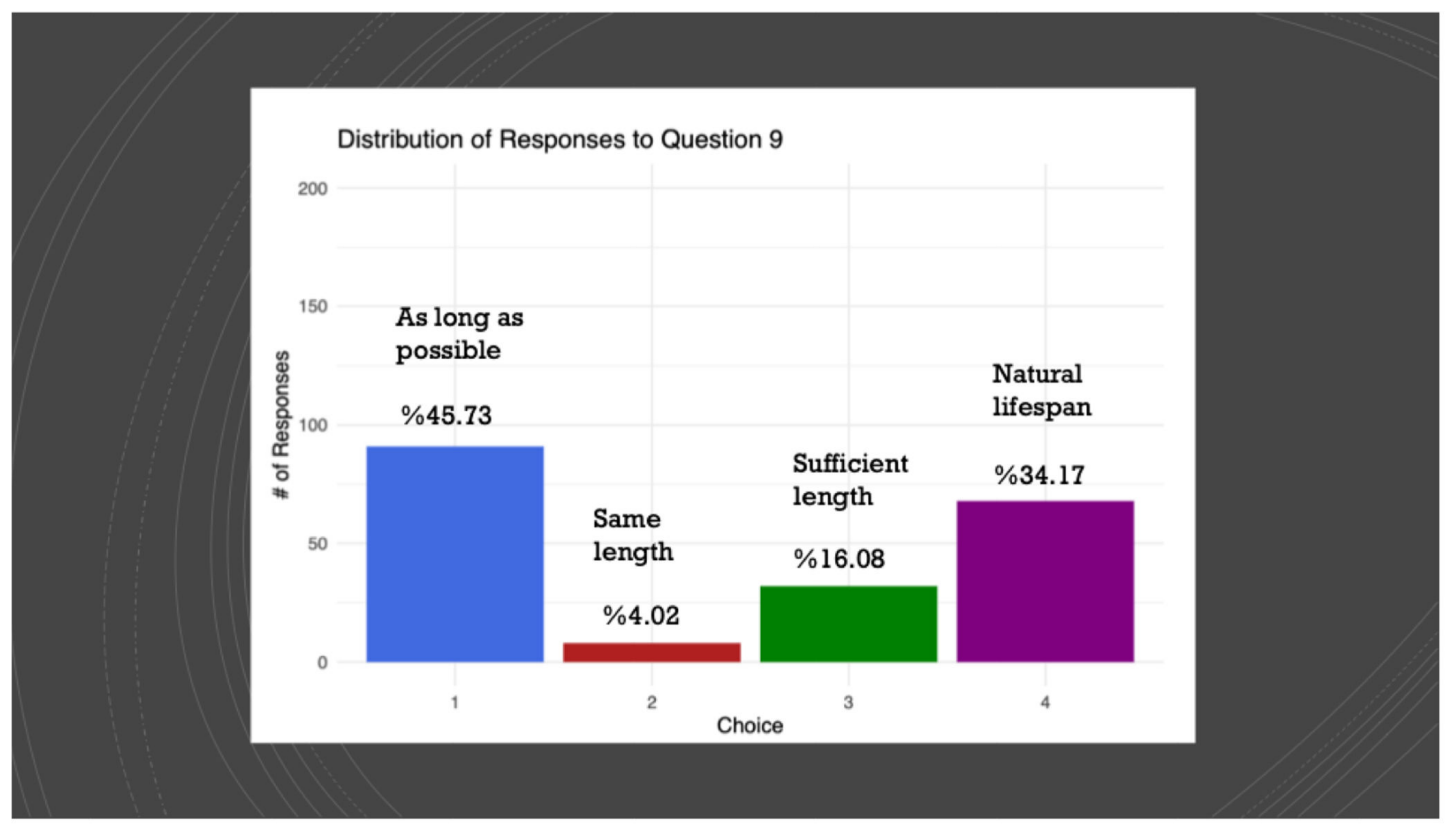


“Sufficient length” was described as “80 years” and “Natural lifespan” was described as 120 years.

Hevolution^61^ is investing billions in developing life extension technologies, or strictly, healthspan extension technologies (but these are likely to also extend life). As life extension technologies mature, we will face profound questions about long people should live. I don't believe we can apply a simple approach, like egalitarianism and a fair innings. These will be important considerations, amongst others. But we require a complex pluralistic approach. We require complex ethics.

## The Speed Limit

I began this piece with the dilemma of who to give a heart transplant to when not everyone can receive one. And I have consider allocation of life-saving resources in the pandemic. I will finish on what many will find is a much more mundane issue: the speed limit. (I know driving is another of John's pleasures − he refuses to buy a driverless car because he enjoys driving so much. I am sure he always keeps to the speed limit.)

As I travel the world and live in different places, I am fascinated that the speed limit is different in each country. In the UK, it is 120kph, in Germany it is unlimited on the autobahn, in France it is 130kph, in Victoria, Australia it is 100kph, in NSW (a neighbouring state of Australia) it is 110kph, and in Singapore it is only 90kph!

Driving a car is a potentially lethal activity. Each year 1.19 million people die on the roads globally (WHO) and speed is a major contributor. So what is the right speed limit?

As I have argued, ethical judgements are context specific. The answer will depend in part on many facts: the quality of the roads, how crowded, the safety of the cars, visibility, weather, orientation of the roads and geography, etc.

But importantly which speed limit is right will depend on values. Safety is one value. The lower the limit, the safer. So on safety grounds, Singapore has picked the right one. Protecting the environment is another important value. A lower limit reduces carbon emissions. So is the lowest limit − 90 kph − the right one?

There are other values. Economic efficiency supports a higher speed limit. The Netherlands recently increased its speed limit to 130kph to increase economic efficiency. Pleasure supports a higher limit. Freedom supports a higher limit. And so on.

An ethical policy will take account of all the relevant values and facts. But it is not that a complete specification of all the relevant facts will directly dictate a policy. Nor will a single value dictate the correct policy. It is complex. What is required is an ethical process and deliberation about how to weigh the different values and apply them to the facts (and wisdom).

Importantly, not any speed limit goes. Setting a limit of 100kph in a crowded city centre with small roads and school crossings is wrong. While there may be some grey zones, that might be informed by cultural or societal values, there is black and white, there is wrong and right. Some speed limits are wrong.

The task of bioethics to create rules for such deliberation, to outline the black, white and grey. Constructing an ethical algorithm is one way of taking account of plural values, including equality, and enabling them to be applied to the facts of the situation.

## Vector of Reasons

Ethics is one way like physics. In Newtonian physics, forces can be described as vectors. They have a direction and strength. Which direction a ball will roll is predicted by summing the vectors.

In ethics, the issue is which direction *should* the ball roll. That is determined by reasons. Reasons are like vectors. They have a direction and a strength, depending on the factual circumstances. For example, in the speed limit case, the vector of safety would be a mere point if cars were perfectly safe for users and third parties. What should be done, the right policy, weighs all these vectors, or reasons.

While we know the laws of physics, we have yet to entirely discover or invent the laws of ethical deliberation. That is, how to identify all relevant reasons, and how to weigh and prioritise them.

Collective reflective equilibrium and algorithmic bioethics are two miniscule steps forwards in the physics of ethics. (Physics began as a discipline within philosophy at the University of Oxford.)

## Conclusion

The point of bioethics and practical ethics is to inform practical decision making and policy. Indeed, ethical decisions are inescapable and to choose to do nothing, including not to think about the problem, is to be responsible for the consequences of that course, when we have the power to choose to act another way. It is inevitable that policies and laws have to be made, like setting a speed limit. We should aim to improve them, and make the best decisions, policies and laws possible, given the constraints of our situation and relevant opportunity costs (for example, of delaying policy making to gather more information, or deliberate more). Just as “first come, first served” is a bad procedure for allocating life saving resources, so too many of the intuitive, or monotheistic, policies may be mistaken.

John Harris is, in my view, one of the great pioneers in bioethics. He has articulated, defended and applied highly plausible accounts of egalitarianism and what is now called sufficientarianism. He has given a healthy and piercing critique of utilitarianism and QALYs. However, bioethics is an ongoing project where progress is incremental and slow. What remains to be done − the task of bioethics - is to place these arguments in a broader complex framework looking for coherence with other plausible theories and principles. Indeed, it will be to create new theories and principles. And, I believe at least for the purposes of public policy, to identify points of maximum coherence with public values. (Of course, it is a further question is what should constitute public values and how we should ascertain these.)

As someone who has been battling in this field now for 35 years, I have frequently used John's arguments as a springboard for debate and progress. I would like to thank John Harris for his contribution to the field.

When I wrote that piece on allocation of organs to children which I began with, I received considerable anger and hostility from the clinical genetics community for daring to raise these issues. I was complaining to John McBain (who established Melbourne IVF) about how unfair I felt people's reactions were. He replied,

“In Scotland, the worst thing you can say about a man is that he died with no enemies.”

In bioethics, the best thing you can say about someone is not that they created a flock of followers like sheep, but that they caused people to think and they enabled progress. John Harris has innervated and energised the field of bioethics with his brand of egalitarianism for over 40 years. That's about all you can do.
